# Novel Glycopolymer Eradicates Antibiotic- and CCCP-Induced Persister Cells in *Pseudomonas aeruginosa*

**DOI:** 10.3389/fmicb.2018.01724

**Published:** 2018-08-03

**Authors:** Vidya P. Narayanaswamy, Laura L. Keagy, Kathryn Duris, William Wiesmann, Allister J. Loughran, Stacy M. Townsend, Shenda Baker

**Affiliations:** Synedgen, Inc., Claremont, CA, United States

**Keywords:** persister cells, chronic infection, glycopolymer, *Pseudomonas aeruginosa*, PAAG

## Abstract

Antibiotic treatments often fail to completely eradicate a bacterial infection, leaving behind an antibiotic-tolerant subpopulation of intact bacterial cells called persisters. Persisters are considered a major cause for treatment failure and are thought to greatly contribute to the recalcitrance of chronic infections. *Pseudomonas aeruginosa* infections are commonly associated with elevated levels of drug-tolerant persister cells, posing a serious threat to human health. This study represents the first time a novel large molecule polycationic glycopolymer, poly (acetyl, arginyl) glucosamine (PAAG), has been evaluated against antibiotic and carbonyl cyanide *m*-chlorophenylhydrazone induced *P. aeruginosa* persisters. PAAG eliminated eliminated persisters at concentrations that show no significant cytotoxicity on human lung epithelial cells. PAAG demonstrated rapid bactericidal activity against both forms of induced *P. aeruginosa* persister cells resulting in complete eradication of the *in vitro* persister cells within 24 h of treatment. PAAG demonstrated greater efficacy against persisters *in vitro* than antibiotics currently being used to treat persistent chronic infections such as tobramycin, colistin, azithromycin, aztreonam, and clarithromycin. PAAG caused rapid permeabilization of the cell membrane and caused significant membrane depolarization in persister cells. PAAG efficacy against these bacterial subpopulations suggests it may have substantial therapeutic potential for eliminating recurrent *P. aeruginosa* infections.

## Introduction

*Pseudomonas aeruginosa* is an opportunistic pathogen that often causes nosocomial infections in immunocompromised patients and is one of the primary agents responsible for pulmonary decline and early mortality in patients with cystic fibrosis (CF; Mendelson et al., 1994; Dunn and Wunderink, 1995; Govan and Deretic, 1996; Gibson et al., 2003). *P. aeruginosa* reaches relatively high densities in the CF lung, and a substantial fraction of the cells present are in a low metabolic activity state correlated with persister cell status (Yang et al., 2008). The frequent use of high doses of bactericidal antibiotics during chronic infections may lead to selective mutations that produce heightened levels of persisters (Keren et al., 2004). Multiple lines of evidence suggest that the recalcitrant nature of *P. aeruginosa* infections in CF lungs is caused by a drug-tolerant subpopulation of persister cells (Burns et al., 1999; Gilligan, 2006; Yang et al., 2008; Mulcahy et al., 2010).

Persisters are a small fraction of non-replicating, metabolically quiescent bacteria tolerant to antibiotic killing (Keren et al., 2004; LaFleur et al., 2010; Mulcahy et al., 2010). These antibiotic-tolerant bacterial cells have a growth-arrested phenotype and are capable of recommencing growth after a stress event (Lewis, 2007, 2008; Kim and Wood, 2016). Due to their state of metabolic dormancy, persisters have a high tolerance against traditional classes of antibiotics such as fluoroquinolones, aminoglycosides, and beta-lactams, which are only effective against metabolically active cells. Antibiotics that are bactericidal against planktonic cells are typically ineffective against persister cells (Hoyle et al., 1990). Once the local antibiotic concentration drops and the nutrients are available (Kim et al., 2018), persisters can become metabolically active again and reestablish the infection (Lewis, 2007, 2008) causing the relapsing chronic infections often observed in CF patients (Lewis, 2008).

The ineffectiveness of conventional systemic antibiotics for treating chronic pulmonary infections have led to treatment with high doses of inhaled antibiotics including azithromycin, aztreonam, and tobramycin (Mearns, 1972; Geller et al., 2002; Zindani et al., 2006). During such treatments, aerosolized tobramycin can reach peak concentrations of 1,237 μg/g of sputum, which is ≥25 times higher than the minimum inhibitory concentration (MIC) of most tested clinical isolates of *P. aeruginosa* (Geller et al., 2002). Inhalation treatments with levofloxacin achieve up to 1,760 μg/g of sputum, a concentration that is >50 times higher than MIC of clinical isolates of *P. aeruginosa* (King et al., 2010). Tobramycin and levofloxacin at these concentrations effectively kill actively growing resistant bacteria but induce a stress event that supports persister cell phenotype development (King et al., 2010; Lewis, 2010). Inhaled tobramycin has long been identified to control but not eradicate *P. aeruginosa* infections in patients with chronic lung infections (Ramsey et al., 1999; Gibson et al., 2003). The decrease in efficacy of tobramycin over treatment time can be attributed to and is consistent with an increase in the numbers of persisters (Koeva et al., 2017).

The limited activity of traditional antibiotics against persisters is due to attenuation of active bacterial transport mechanisms along with low metabolic rates (Davis, 1987; Allison et al., 2011). Metabolite stimulation of the proton motive force (PMF) has been shown to awaken the cells (Kim et al., 2018) therefore improve the uptake of aminoglycosides and increase effectiveness of bacterial persister killing, helping to clear the infection (Allison et al., 2011; Koeva et al., 2017). Fructose in combination with gentamicin was observed to be effective against *Escherichia coli* as well as *Staphylococcus aureus* persisters (Lebeaux et al., 2014). Metabolite enabled killing of *P. aeruginosa* persisters has been observed with aminoglycosides and fructose as well as mannitol and tobramycin (Barraud et al., 2013; Orman and Brynildsen, 2013). Arginine and nitrate were also described as useful additives in improving the uptake of aminoglycosides (Borriello et al., 2006). A recently published study suggests a fumarate antibacterial potentiator used in combination with tobramycin enhanced killing of persister cells compared to tobramycin alone (Koeva et al., 2017). Together these studies show the influence of bacterial transport mechanisms and metabolism on antibiotic tolerance exhibited by persister cells.

Another potential class of antibacterial used in persister treatment and research are antimicrobial peptides (AMPs). AMPs are known for their ability to disrupt bacterial membranes and/or translocate across membranes to bind intracellular targets. A major drawback to this approach is the potential toxicity to mammalian cells (Zhao et al., 2013; Kang et al., 2014). AMPs potential to disrupt bacterial membranes is less dependent on the metabolic status of the bacterial cell (Hurdle et al., 2011). Colistin disrupts the outer membrane of Gram-negative species and is bactericidal against persisters (Cui et al., 2016), but the concentration required to eradicate persisters is above clinically acceptable concentrations, leading to toxicity, including renal and neurological side effects (Cui et al., 2016). Despite major progress in understanding the metabolic changes and mechanisms of bacterial persister cell formation, several impediments to clinical applications remain (Barraud et al., 2013; Chowdhury et al., 2016; Wood, 2017). The observation that membrane-disrupting antibiotics can kill persisters is an important discovery that suggests a therapeutic way forward.

Poly (acetyl, arginyl) glucosamine (PAAG), is among a novel class of polysaccharide therapeutics with antimicrobial activity against a wide range of pathogenic Gram-negative and Gram-positive bacterial species including methicillin-resistant *Staphylococcus aureus* (MRSA) (Narayanaswamy et al., 2018), multiple species of *Burkholderia* (Narayanaswamy et al., 2017), *E. coli* (Tang et al., 2010), and non-tuberculous *Mycobacteria* (NTM; Unpublished data). The antimicrobial efficacy of PAAG has been attributed to its polycationic nature (Tang et al., 2010; Narayanaswamy et al., 2017). PAAG rapidly permeabilizes Gram negative bacteria (Tang et al., 2010; Narayanaswamy et al., 2017) at the doses that we report in this study, which have minimal cytotoxic effects *in vitro* when tested against the human epithelial lung cell line, A549.

Aims of the present study were to (i) evaluate the ability of PAAG to eradicate antibiotic-induced *P. aeruginosa* persisters, commonly implicated in relapsing and chronic lung infections, (ii) evaluate the susceptibility of carbonyl cyanide *m*-chlorophenylhydrazone (CCCP)-induced persisters to membrane active agents like colistin and polycationic glycopolymer PAAG, (iii) characterize the membrane activity of PAAG and other antibiotics on *P. aeruginosa* persister-like cells in the presence and absence of CCCP, and (iv) test the cytotoxic effects of PAAG on A549 lung epithelial cells.

## Materials and Methods

### Bacterial Strains, Reagents, and Growth Conditions

The bacterial strain used in the study was *P. aeruginosa* PA01 (a kind donation from Paul Orwin, CSUSB). Bacteria were initially streaked from -80°C glycerol stock onto Luria–Bertani agar (LB Agar; Difco, Fisher Scientific) plates and incubated at 37°C overnight. Minimal BM2 medium (Poole et al., 1991; Reffuveille et al., 2014; Mlynarcik and Kolar, 2017) with glucose as the carbon source [phosphate ammonia base (0.07 M (NH_4_)_2_SO_4_ – 9.25 g, 0.4 M K_2_HPO_4_ – 69.7 g, 0.22 KH_2_PO_4_ – 29.9 g, pH 7.0), 50 mM MgSO_4_, 10 mM FeSO_4_, and 40% glucose] and LB broth was used to culture *P. aeruginosa* cells. Planktonic stationary-phase and exponential cultures were prepared as previously described (40). Planktonic stationary-phase cultures were prepared by inoculating from a fresh LB agar plate into 5 mL LB broth (Difco, Fisher Scientific) and incubating them in a shaker incubator overnight at 37°C. For experiments, 1 mL of the overnight culture was centrifuged at 13,000 rpm for 1 min, then washed and resuspended in BM2 glucose media. Planktonic exponential-phase cultures were prepared by inoculating 10^7^ cells/mL in BM2 glucose media and growing them to an optical density (600 nm) of 0.6–0.8. The bacterial cells were exposed to PAAG (Synedgen, Inc.), azithromycin (TCI), tobramycin (Sigma), clarithromycin (Fluka), aztreonam (TCI), and colistin (TCI) at the concentrations and time periods stated throughout the article. CCCP was purchased from Sigma-Aldrich. Stock solution of 0.2 mg/mL CCCP was diluted in DMSO and stored as aliquots at -20°C.

### Antimicrobial Susceptibility Assay

Sterile aqueous stock solutions of PAAG (10–1,000 μg/mL), azithromycin (8–512 μg/mL), ciprofloxacin (0.25–64 μg/mL), ofloxacin (0.25–64 μg/mL), clarithromycin (8–512 μg/mL), tobramycin (2–128 μg/mL), colistin (8–512 μg/mL), aztreonam (8–512 μg/mL), and CCCP (200 μg/mL) were used to perform MIC) assays. Serial dilutions of each antibiotic/PAAG were prepared in the presence or absence of CCCP in 50 μL Mueller Hinton Broth (MHB) in 96-well microtiter plates. Exponential phase broth culture of *P. aeruginosa* PA01, diluted to a cell density of 1.5 × 10^8^ CFU/mL, were verified by total viable count. Then 50 μL of the inoculum is added to each well of the microtiter plates. Positive and negative growth controls were included in each plate to verify the test method. Plates were incubated at 37°C and MIC values were determined for each antimicrobial as the lowest concentration at which growth was inhibited. The experiment was performed in triplicate with three independent cultures.

### Time Kill Assay

Planktonic stationary phase cultures were prepared as described above. Briefly overnight cultures of *P. aeruginosa* cells were pelleted (13,000 rpm for 1 min), washed and resuspended in BM2 glucose media. An inoculum of 20 μL *P. aeruginosa* suspension was added into 180 μL of BM2 glucose media containing concentrations of the antimicrobials, four times their MIC [PAAG (100–200 μg/mL), tobramycin (16 μg/mL), clarithromycin (256 μg/mL), colistin (256 μg/mL), aztreonam (256 μg/mL), azithromycin (256 μg/mL)] in 96-well plates. The plates were sealed with Parafilm, loaded onto microplate shakers, and placed in a 37°C incubator for 10, 30 min and 1, 2, 4, 8, 24 h. At each time point, 10-fold serial dilutions were made with BM2 glucose media and the dilutions were spot plated onto LB agar plates and incubated overnight at 37°C to yield visible colonies. The experiments were performed in triplicate with three independent cultures.

### CCCP-Induced Persisters

Pretreatment with CCCP at a concentration of 200 μg/mL for 3 h has been observed to induce a 5,000-fold increase in the number of persister-like cells in cultures subsequently exposed to antibiotics (Grassi et al., 2017). The concentration of CCCP and the treatment time required to induce persistence in stationary-phase cultures of *P. aeruginosa* has been previously described by Grassi et al. (2017). Overnight cultures of *P. aeruginosa* were incubated with 200 μg/mL of CCCP for 3 h at 37°C with shaking. Following pretreatment with CCCP, bacteria were washed twice in BM2 glucose media, pelleted (13,000 rpm for 1 min), and re-suspended in BM2 glucose media to a final density of 1.5 × 10^8^ CFU/mL. Bacterial suspensions induced with CCCP were exposed to the following antibiotics at concentrations four times the MIC: tobramycin (16 μg/mL), clarithromycin (256 μg/mL), aztreonam (256 μg/mL), azithromycin (256 μg/mL). Untreated and CCCP-pretreated bacteria were used as viability controls. The surviving persister cells were determined by CFU counting at 10, 30 min and 1, 2, 4, 8, 24 h of incubation at 37°C. The experiment was performed in triplicate with three independent cultures.

### Effect of PAAG on CCCP-, Antibiotic-Induced Persister Cells

To test the ability of PAAG or colistin to potentiate the effect of antibiotics and thereby eliminate the surviving population of antibiotic-induced persister cells, planktonic stationary phase cultures were exposed to antibiotics [tobramycin (16 μg/mL), clarithromycin (256 μg/mL), or aztreonam (256 μg/mL)] and placed in a 37°C incubator for 3 h prior to treatment with PAAG (200 μg/mL) or colistin (256 μg/mL).

CCCP-induced persisters were generated by pretreating *P. aeruginosa* cultures to CCCP (200 μg/mL) for 3 h at 37°C as described above. Briefly, the CCCP-induced persister cells were pelleted and resuspended in BM2 glucose media and 20 μL was added to wells containing 180 μL of PAAG or colistin at a concentration of 100–200 or 256 μg/mL respectively.

The plates were sealed with Parafilm, loaded onto microplate shakers in a 37°C incubator for 24 h. At 30 min and 1, 2, 4, 8, 24 h 10-fold dilutions were made with BM2 glucose media and spot plated onto LB plates to determine CFU/mL. The experiment was performed in triplicate with three biological replicates.

### Propidium Iodide (PI) Uptake Assay

CCCP-induced cultures of *P. aeruginosa* were generated as mentioned above and were treated with antibiotics [tobramycin (16 μg/mL), clarithromycin (256 μg/mL), colistin (256 μg/mL), aztreonam (256 μg/mL), azithromycin (256 μg/mL), or PAAG (100–200 μg/mL)] for 3 h, pelleted, washed and resuspended in BM2 glucose media. PI was used at a concentration of 17 μg/mL as described in previous studies (Kwan et al., 2015). All assays were performed at room temperature. PI at a concentration of 17 μg/mL was added to the wells of a 96 well plate containing 1.5 × 10^8^ CFU/mL of the prepared bacterial culture and fluorescence was measured via SpectraMax Gemini XPS (Molecular Devices). PAAG (100 and 200 μg/mL), ciprofloxacin (2 μg/mL), ampicillin (100 μg/mL), tobramycin (16 μg/mL), clarithromycin (256 μg/mL), colistin (256 μg/mL), aztreonam (256 μg/mL), azithromycin (256 μg/mL) were added to the wells containing the mixture. Cells treated with 0.1% Triton X-100 was used as a positive control. PI alone and PI on cells were used as negative controls. The mixture was mixed thoroughly prior to obtaining fluorescence measurements. Fluorescence was measured at excitation and emission wavelengths of 535 and 625 nm, every 10 min up to 4 h. The experiment was performed in triplicate with three independent cultures.

### Cytoplasmic Membrane Depolarization Assay

The membrane depolarization activity of PAAG and colistin was determined using the membrane potential-sensitive fluorescent dye 3,3′-Dipropylthiadicarbocyanine iodide (DiSC3-5) (Suzuki et al., 2003). CCCP-induced persister cells of *P. aeruginosa* PA01 were grown as described above and subsequently treated with tobramycin (16 μg/mL) for 3 h at 37°C. The cells were collected by centrifugation and washed three times with 5 mM HEPES (pH 7.8) buffer. The cells were then resuspended in 5 mM HEPES (pH 7.8) buffer with 0.2 mM EDTA to an OD600 of 0.05 (45). DiSC3(5) and KCl was added at the final concentration of 0.4 uM and 0.1 M, respectively, and incubated at 37°C shaker (150 rpm) incubator for 20 min until the fluorescence quenching was achieved. PAAG and colistin at a final concentration of 50–200 and 256 μg/mL, respectively, was added, and the fluorescence was monitored under shaking conditions at 37°C at an excitation wavelength of 622 nm and an emission wavelength of 670 nm subsequently after 15 min of incubation. Wells with cells and the dye were used as background. Triton X-100 (0.1% v/v), a commonly used membrane disruptor, was used as the positive control (Saar et al., 2005). The experiment was performed in triplicate with three independent cultures.

### Cytotoxicity Assay

The cytotoxicity of PAAG was investigated on A549 cells (ATCC CRM-CCL-185) using the Pierce lactate dehydrogenase (LDH) Cytotoxicity Kit (Promega, Madison, WI,United States). Cells were seeded in a 96-well plate at a concentration of 2.0 × 10^4^ cells/well. Then, 100 μL of F12K media was added to each well-containing varying concentrations of PAAG (65–1,000 μg/mL) and incubated for 24 h. Briefly, 50 μL of media was transferred from each treatment well to a new 96-well plate, then mixed with 50 μL of LDH reaction mixture and incubated at room temperature for 30 min. After incubation, 50 μL of stop solution was added and the plate was read using a wavelength of 490 and 680 nm on a microplate reader (SpectraMax Gemini XPS; Molecular Devices). The untreated cell population represents the baseline levels of LDH from A549 cells and a lysis buffer control (provided in the kit) was used to normalize the data. 10× lysis buffer (provided by the manufacturer) was used as the positive control. The % cytotoxicity was calculated according to the manufacturer’s instructions. Briefly, % cytotoxicity equals the difference between the PAAG-treaded LDH activity and the Spontaneous LDH activity (water-treated control), divided by the difference of the maximum LDH activity (lysis buffer) and the spontaneous LDH activity, multiplied by 100. Data represented as % viability. The experiment was performed in triplicate.

### Statistical Analysis

All the *in vitro* experiments were performed in triplicate. The statistical analysis was conducted using GraphPad Prism 6.0 (GraphPad Software, Inc., San Diego, CA, United States). Results are expressed as means ± standard error of the mean (SEM).

## Results

### Antibacterial Activity of the Antimicrobials Against Planktonic Cells

The MIC’s of PAAG, tobramycin (TOB), azithromycin (AZI), clarithromycin (CLA), aztreonam (ATM), ofloxacin (OFX), ciprofloxacin (CIP), and colistin (CST) were determined to be 30, 4, 64, 64, 64, 0.5, 0.5, and 64 μg/mL, respectively, as shown in **Table 1**. The CSLI standard clinical breakpoints are also noted (CLSI, 2017). As shown in **Table 1**, presence of CCCP resulted in substantial reduction in the MIC of colistin. Though the MIC’s of other antibiotics showed a slight increase in MIC in the presence of CCCP (**Table 1**).

**Table 1:** Antibiotic susceptibility of planktonic *P. aeruginosa* PA01 to antimicrobials.

*P. aeruginosa* PA01	MIC (μg/mL)							
	PAAG	CST	TOB	ATM	AZI	CLA	OFX	CIP
	30 [30]	64 (R) [4 (S)]	4 (S) [8 (I)]	64 (R) [128 (R)]	64 [128]	64 [64]	0.5 (S) [0.5 (S)]	0.5 (S) [0.25 (S)]

*Clinical breakpoints for antibiotics according to CSLI standards were ≤2 μg/mL (S), ≥4 μg/mL (R) for colistin (CST); ≥16 μg/mL (R), 8 μg/mL (I), ≤4 μg/mL (S) for tobramycin (TOB); ≥32 μg/mL (R), 16 μg/mL (I), ≤8 μg/mL (S) for aztreonam (ATM); ≥8 μg/mL (R), 4 μg/mL (I), ≤2 μg/mL (S) for ofloxacin (OFX); ≥4 μg/mL (R), 2 μg/mL (I), ≤1 μg/mL (S) for ciprofloxacin (CIP). R, resistant strains; S, Sensitive strains; I, Intermediate. MIC’s in [] represent were taken in the presence of CCCP.*

### Bactericidal Effects of Antimicrobials

**FIGURE 1:**
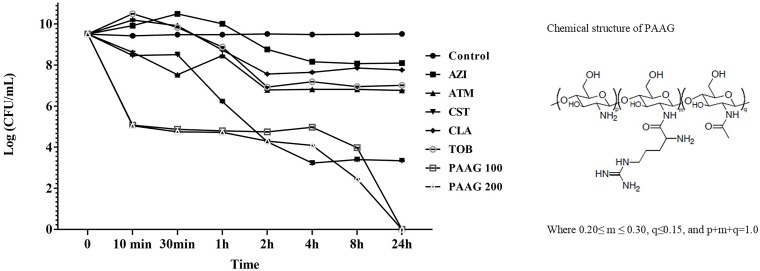
PAAG kills stationary phase *Pseudomonas aeruginosa* cultures without forming persisters. Stationary phase *P. aeruginosa* cultures were challenged with tobramycin (16 μg/mL), clarithromycin (256 μg/mL), colistin (256 μg/mL), aztreonam (256 μg/mL), azithromycin (256 μg/mL), or PAAG (100–200 μg/mL) for 24 h. Samples were aliquoted and plated for viable plate count (CFU/mL) at multiple timepoints over 24 h. Unchallenged control samples were aliquoted directly prior to antibiotic treatment. Data represents average of three independent experiments, and the error bars indicate the standard error of the mean.

Kill curves for each antibiotic were assessed to identify the antibiotic concentrations enabling survival of a small drug-tolerant subpopulation (**Figure 1**). Antibiotics like tobramycin, and aztreonam exhibited a 3-log reduction in CFU/mL. Azithromycin and clarithromycin displayed 2-log reduction in CFU/mL. The afore mentioned antibiotics demonstrated limited bactericidal activity even at concentrations four times its MIC indicative of limited bactericidal activity against stationary phase cultures of *P. aeruginosa*. Colistin, as a cell wall disruptor, demonstrated potent bactericidal activity against stationary phase cultures, resulting in a 5-log reduction in CFU/mL after 3 h of treatment (*P* < 0.001; **Figure 1**). Treatment with PAAG at concentrations of 100 and 200 μg/mL, resulted in a 6-log reduction in CFU/mL compared to the untreated control within 10 min (*P* < 0.001) and total eradication by 24 h.

At concentrations four times their MIC, all the antibiotics except PAAG tested demonstrated a biphasic time-kill curve with a slow decrease in CFU/mL of stationary phase cultures over the first 3 h of treatment, followed by stabilization of the viable population at 24 h (**Figure 1**).

### PAAG Potentiates Killing of Antibiotic-Induced Persisters

**FIGURE 2:**
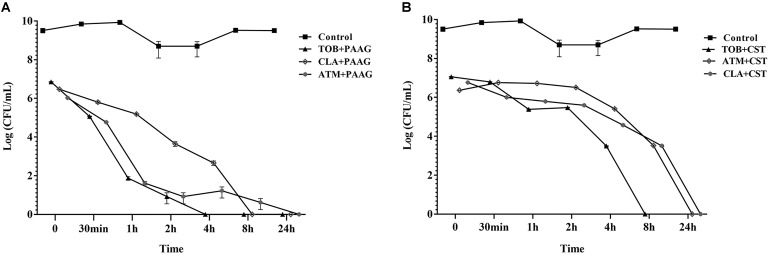
**(A,B)**. Effect of antimicrobials against antibiotic- induced *P. aeruginosa* persister cells. Activity of PAAG and colistin at a concentration of 200 and 256 μg/mL, respectively, was assessed against stationary phase cultures of *P. aeruginosa* PA01 pretreated with tobramycin (16 μg/mL), aztreonam (256 μg/mL), or clarithromycin (256 μg/mL) for 3 h. After the addition of PAAG or colistin samples were aliquoted and plated for colony count (CFU/mL) at multiple time points over 24 h. Non-challenged control samples were plated immediately prior to PAAG treatment. T = 0 is 3 h post-antibiotic treatment. Data represents average ± standard error of the mean of three independent experiments.

Antibiotic tolerant cells were isolated by treating stationary phase cultures of *P. aeruginosa* with tobramycin, aztreonam or clarithromycin as described in Section “Materials and Methods.” The surviving antibiotic tolerant bacteria were further challenged with PAAG or colistin at a concentration of 200 and 256 μg/mL respectively (**Figure 2**). Treatment with PAAG demonstrated a 6- to 7-log reduction (CFU/mL) within 2–4 h of treatment and complete eradication within 24 h (**Figure 2A**). Colistin at concentrations 4× MIC was observed to be bactericidal (3- to 4-log reduction) against the antibiotic induced persister population in 2 h of treatment and resulted in complete eradication of the antibiotic tolerant population of the *P. aeruginosa* cells in 8–24 h of treatment (**Figure 2B**).

### Induction of Persistence in the Presence of CCCP

**FIGURE 3:**
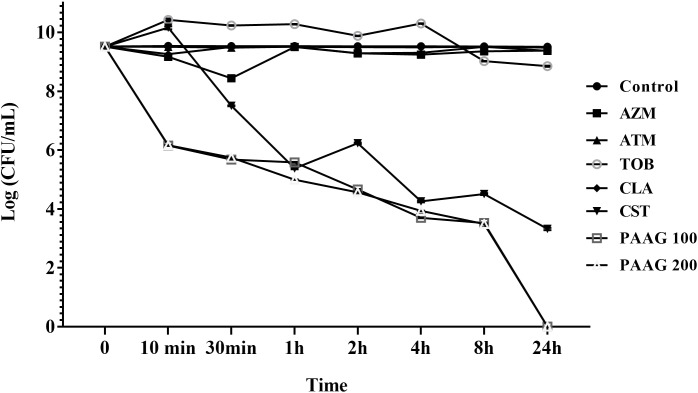
Effect of CCCP to enhance persistence in stationary phase cultures of *P. aeruginosa*. CCCP at a concentration of 200 μg/mL was used to induce persistence was assessed by evaluating the log reduction of cells surviving the treatment with PAAG (100–200 μg/mL) tobramycin (16 μg/mL), clarithromycin (256 μg/mL), colistin (256 μg/mL), aztreonam (256 μg/mL), azithromycin (256 μg/mL) following 3 h exposure to CCCP. Data are reported as mean ± standard error of the mean of the mean of at least three independent experiments.

The ability of CCCP to induce persistence in *P. aeruginosa* cells was evaluated by exposing CCCP-pretreated cultures to different classes of antibiotics with diverse mechanisms of action such as fluoroquinolones, namely the monobactam (aztreonam), aminoglycoside (tobramycin), and macrolide (clarithromycin and azithromycin) as previously described (Grassi et al., 2017). Pretreatment with CCCP (200 μg/mL) for 3 h resulted in significant increase in the survival of *P. aeruginosa* PA01 cells against the different classes of antibiotics. As shown in **Figure 3**, stationary-phase cultures pretreated with CCCP for 3 h demonstrated tolerance to treatment with antibiotics namely aztreonam, azithromycin, clarithromycin and tobramycin as compared to the untreated controls. Pretreatment with CCCP increased the surviving cells to the afore mentioned antibiotic treatments by 67–73% compared to the CCCP-untreated where ∼1.7% of the population survived the antibiotic exposure. On the other hand, treatment of *P. aeruginosa* cultures with CCCP (200 μg/mL) lead to a 7-log decrease in the number of bacteria tolerant to colistin, leaving a small subpopulation of persister cells behind (0.1%). Similarly, treatment with PAAG resulted in 4-log reduction of the bacteria within 1 h of treatment (**Figure 3**). Unlike colistin, PAAG completely eradicated the CCCP-pretreated *P. aeruginosa* cells in 24 h of treatment.

### Effect of PAAG on CCCP-Induced Persisters

**FIGURE 4:**
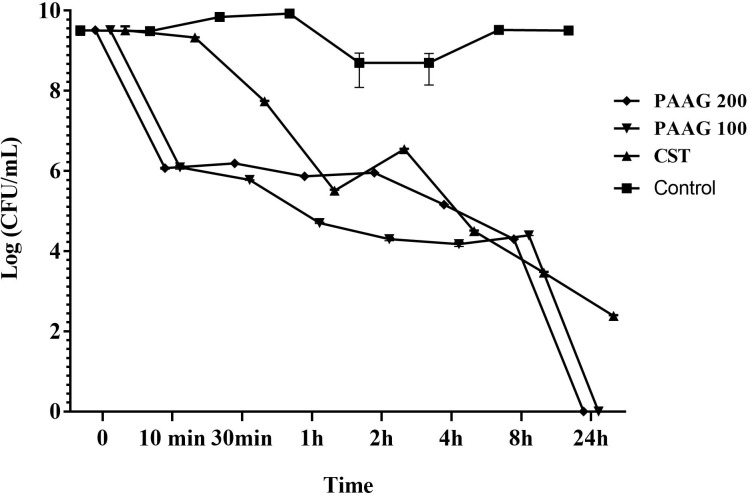
Effect of PAAG compared to a membrane active antibiotic against CCCP induced persisters of *P. aeruginosa* PA01. Activity of PAAG at concentrations of 200 and 100 μg/mL and colistin (256 μg/mL) against CCCP-induced *P. aeruginosa* PA01 cells pretreated with tobramycin (16 μg/mL). Anti-persister activity of PAAG was assessed by CFU counting at 10, 30 min and 1, 2, 4, 8, 24 h. Data represented as average values from three independent experiments ± standard error of the mean.

PAAG’s anti-persister activity was also evaluated based on its competence in eliminating persister-like cells formed in CCCP pretreated *P. aeruginosa* cultures. PAAG displayed bactericidal activity against CCCP-induced persister cells of *P. aeruginosa* and achieved complete killing of the initial bacterial inoculum at concentrations as low as 100 μg/mL (**Figure 4**). Colistin on the other hand was able to eliminate most of the CCCP-induced persisters of *P. aeruginosa* within 24 h of treatment, however, it was observed to leave a sub population of tolerant cells behind even at concentrations 4× its MIC.

### Permeabilization of Persisters

**FIGURE 5:**
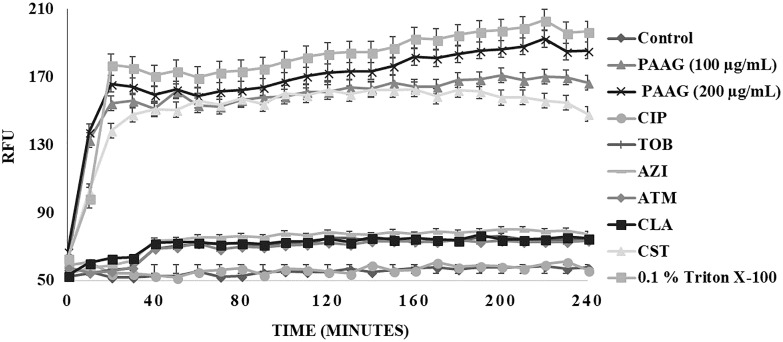
Membrane permeabilization of CCCP- induced *P. aeruginosa* persister cells using propidium iodide (PI) through spectrophotometry. Permeabilization of persister cells formed in stationary phase cultures of *P. aeruginosa* cultures were treated with ciprofloxacin (100 μg/mL), tobramycin (16 μg/mL), colistin (256 μg/mL), azithromycin (256 μg/mL), aztreonam (256 μg/mL), and clarithromycin (256 μg/mL) or PAAG (100–200 μg/mL) and measured by spectrophotometry over 4 h. PI fluorescence was measured pre- and post-treatment with antibiotics or PAAG. CCCP-induced cells with PI was used as a baseline control. The experiment was repeated with there independent cultures and the data is represented as the average ±standard error of the mean.

Propidium iodide (PI) uptake assay was used to measure the ability of PAAG and the other antimicrobials used in this study to permeabilize CCCP-induced persister cells. PI shifts emission upon entry into bacteria and subsequent intercalation into the bacterial DNA. Cells exposed to PI in the absence of antibiotics were used to form a baseline PI fluorescence (**Figure 5**). No significant change in fluorescent intensities was observed upon treating the persister cells with ciprofloxacin (1 μg/mL), tobramycin (16 μg/mL), azithromycin (256 μg/mL), aztreonam (256 μg/mL), and clarithromycin (256 μg/mL) (**Figure 5**). Even after 4 h of incubation, the antibiotics were unable to permeabilize the persister cells (**Figure 5**). Treatment with colistin (256 μg/mL), a membrane active antibiotic, resulted in permeabilization of the persister cells as indicated by corresponding increase in fluorescence intensity. A steady increase in fluorescence intensity was detected with increasing PAAG concentrations (100–200 μg/mL) within 10 min of treatment when compared to the control (**Figure 5**). Both concentrations of PAAG tested showed the ability to permeabilize the bacteria to a comparable level to colistin.

### Membrane Depolarization Assay

**FIGURE 6:**
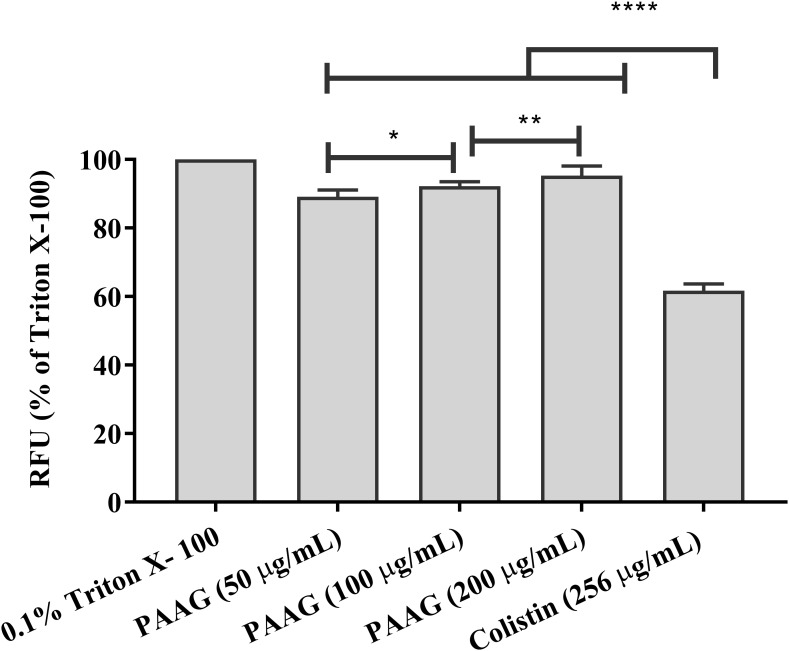
PAAG upon permeabilization causes depolarization of the cytoplasmic membrane. Effect of PAAG or colistin on the cytoplasmic membrane of CCCP-induced *P. aeruginosa* persister cells incubated with DiSC3(5). Results expressed in relative fluorescence units (RFU) observed at 670 nm as compared to negative control. 0.1% Triton X-100 was used as positive control. Cells with DiSC3(5) was used as the baseline control. Data represented as mean ± SEM of three determinations. ^∗∗∗∗^*P* < 0.001, ^∗∗^*P* < 0.01, ^∗^*P* < 0.05. The lines were used to compare the statistical significance between the different concentration of PAAG treatments.

Persister cells, although metabolically different from planktonic cells, are expected to remain susceptible to membrane active agents (Ouberai et al., 2011). At concentrations of 50–200 μg/mL PAAG was able to depolarize the membrane of *P. aeruginosa* persister cells within 10 min of treatment (**Figure 6**). After a 15 min stabilization period, addition of PAAG at concentrations of 50–200 μg/mL resulted in a dose dependent increase in fluorescence intensity corresponding to the change in membrane potential. PAAG at a concentration of 200 μg/mL lead to 91% increase in fluorescent intensity compared to the control, which was statistically more than colistin at 4× its MIC which resulted in a 60% increase in fluorescence intensity.

### Cytotoxicity Assay

**FIGURE 7:**
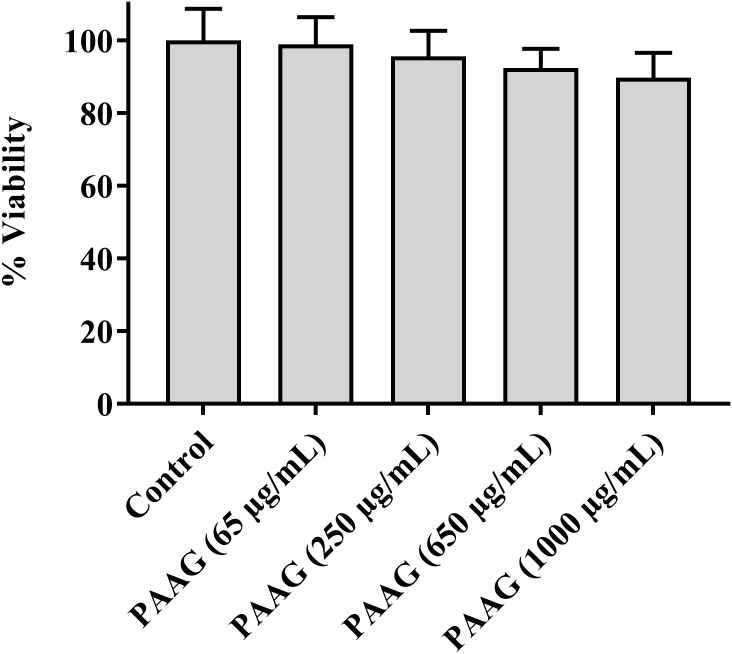
Cytotoxicity assay showing the percent cell viability after incubating A549 cells with PAAG for 24 h. A549 cells were plated onto 96-well plate at an initial seeding density of 2.0 × 10^4^ cells/well. PAAG was dissolved in serum-free F12K media at concentrations ranging from 65 to 1,000 μg/mL. The plates were incubated for 24 h and compound mediated cytotoxicity was determined through pierce LDH cytotoxicity assay. No significant difference compared to the control (Untreated A549 cells). 10× lysis buffer (provided by the manufacturer) was used as the positive control. Data represented as mean ± standard error of the mean. The experiment was performed in triplicate (*n* = 5).

Cytotoxicity of the PAAG glycopolymer was assessed by investigating its effect on A549 cells using an LDH pierce cytotoxicity assay. LDH, a stable cytoplasmic enzyme present in all cells, is rapidly released into the cell culture supernatant when the plasma membrane is damaged. The results indicated that at the concentration range used in this study (65–250 μg/mL), PAAG demonstrated minimal cytotoxic effects (**Figure 7**).

## Discussion

The importance of persister cells in chronic relapsing infections has been emphasized over recent years and has resulted in an increased body of research focused on elucidating strategies aimed at the eradication of these tolerant bacteria (Crunkhorn, 2018; de Breij et al., 2018). Several novel approaches have been suggested for the elimination of these persistent cells, including reverting the phenotype of the bacteria back to a metabolically active state and therefore making it more susceptible to antibiotics by means of chemical compounds or sugars exposure (Pan et al., 2012; Marques et al., 2014; Zhang, 2014). However, identification of antimicrobial agents with a mechanism of action capable of targeting both actively dividing and persistent cells remains an important unmet need. Undeniably, eradication of entire bacterial populations is necessary to avoid the relapse of an infection and thereby minimize minimizing the chance of developing antibiotic resistance during protracted antibiotic treatments (Shan et al., 2017). The current study evaluates the impact of a novel glycopolymer, PAAG, against *P. aeruginosa*, a prototypic persister-forming, bacterial pathogen. Herein commonly used conventional classes of antibiotics such as β-lactams, aminoglycosides, macrolides, and polymyxin are compared to a novel large-molecule glycopolymer.

Antibiotic mechanism of action and varied target specificity are important for selecting the right agent for different clinical settings. However, even though antibiotics such as tobramycin (aminoglycoside), azithromycin and clarithromycin (macrolides), and aztreonam (β-lactam) target different bacterial pathways, they all share the requirement for the bacteria to be metabolically active. Bacterial persistence is a survival mechanism bacterium can employ to avoid killing by multiple classes of antibiotics without the need for genetically encoded resistance. The necessity for active growth was displayed in the observation of reduced antibiotic activity of the afore mentioned agents compared to PAAG, only 2–3 log reduction in bacterial counts, against stationary phase, non-replicating, cultures of *P. aeruginosa* (**Figure 1**). Bacteria treated with these antibiotics at concentrations four times their MIC resulted in a population of persistent cells (**Figure 1**). The reduced metabolic activity of stationary phase cells coupled with the stress of the antibiotics is the accepted mechanism for driving the bacteria into a persistent state (Cabral et al., 2018). However, colistin, a polymyxin, disrupts bacterial membranes to kill *P. aeruginosa*, whether or not actively growing. The increased activity of colistin against dormant cells was shown by the significant reduction of the stationary phase cultures of *P. aeruginosa* (CFU/mL) compared to other conventional antibiotics tested (**Figure 1**). However, colistin alone was still unable to completely eradicate the stationary phase cultures of *P. aeruginosa*. Conversely, both concentrations of PAAG tested were able to completely eradicate stationary phase cultures of *P. aeruginosa* within 24 h (9-logs), also shown in **Figure 1**. Due the slow growth of persisters and the need to become active again to grow, PAAG killing was monitored until 72 h post-treatment at which point there was still not growth observed.

Excessive antibiotic treatment can drive persister formation and lead to recurrence of infection once the local antibiotic concentration has dropped. To evaluate PAAG’s activity in potentiating the antibiotics against antibiotic-induced persistent bacteria, *P. aeruginosa* was first treated with either tobramycin, aztreonam or clarithromycin, all at 4× their tested MICs (**Table 1**) to eradicate the sensitive bulk of the population. No significant difference in killing was observed beyond concentrations 4× MIC (data not shown). The resultant subpopulation of cells tolerant to the antibiotic treatment (antibiotic-induced persistent bacteria) were then treated with PAAG or colistin at a concentration of 200 or 256 μg/mL, respectively (**Figure 2**) in addition to the other antibiotics. Treatment with PAAG, like colistin, resulted in eradication (6- to 7-log reduction) of the antibiotic-induced persistent populations of *P. aeruginosa*. This result suggests that PAAG is not only able kill the bacteria by itself but observed to potentiate the current antibiotics in treating *P. aeruginosa* similar to previous observations with *S. aureus* and *Burkholderia* (Narayanaswamy et al., 2017).

Persister studies are frequently complicated by low levels of naturally forming persisters in antibiotic-based methods and difficulties in separating the surviving persister subpopulation from the substantial proportion of dead bacteria. Consequently, CCCP has been used to enhance generation of persisters (Kwan et al., 2013; Grassi et al., 2017). The development of a persister-like status in CCCP-treated cells has been linked to the inhibition of ATP synthesis and the consequent drop in their metabolic activity (Kwan et al., 2013). The CCCP model system forms persisters through ATP depletion, recently demonstrated in *S. aureus, P. aeruginosa*, and *E. coli* (Davis, 1987; Morita et al., 2012; Grassi et al., 2017). CCCP-induced persisters would be expected to show increased tolerance against antibiotics that required metabolic activity to function. Indeed, pretreatment with CCCP was observed to protect *P. aeruginosa* cells from killing by tobramycin, azithromycin, clarithromycin, and aztreonam (**Figure 3**). Colistin treatment, on the other hand, lead to a 5-log reduction of the CCCP-pretreated persister cells due to its ability to kill growth-arrested bacteria (**Figure 3**). Colistin has been previously shown to demonstrate better bactericidal activity in the presence of CCCP than alone, because treatment with CCCP was found to alter the charge of the lipopolysaccharide (LPS) of the bacteria making them more susceptible to colistin (Pamp et al., 2008; Fernández et al., 2013). Treatment with PAAG, like colistin, resulted in significant reduction of the CCCP-induced persister cells. However, unlike colistin that only had a 5-log reduction, both concentrations of PAAG (100 and 200 μg/mL) eradicated 9-logs of persisters within 24 h (**Figure 3**).

Colistin is a polycationic, amphiphilic peptide that interacts with the negative charges of the lipid A portion of LPS and intercalates into the membrane, causing bacterial permeabilization and cell death (Pamp et al., 2008; Fernández et al., 2013). As a polycationic glycopolymer, PAAG has been shown to permeabilize *S. aureus*, a Gram-positive bacterium (Narayanaswamy et al., 2017), to synergize antibiotics against *Burkholderia* strains (Narayanaswamy et al., 2017) and is hypothesized to also target the negative charges of the LPS similar to colistin. The ability of PAAG to also kill metabolically inactive persister cells suggested that PAAG’s mechanism of action also disrupted *P. aeruginosa* bacterial membranes leading to permeabilization. To investigate PAAG permeabilization of the bacteria, a propidium-iodide (PI) assay was used. PI can only intercalate into the DNA if the bacteria are permeabilized or become permeable overtime as they die. As expected, the control and cytoplasmic active antibiotics (ciprofloxacin, tobramycin, azithromycin, aztreonam, and clarithromycin) did not lead to permeabilization and did not cause significant death (**Figure 5**). In contrast, the rapid increase in the fluorescence intensity shown in **Figure 5** indicated that both colistin and PAAG permeabilized the CCCP-induced *P. aeruginosa* persister cells rapidly. To determine the extent to which PAAG leads to disruption of the bacterial membranes and to compare its activity with colistin, a membrane depolarization assay was used. Cytoplasmic membrane depolarization was assessed using a membrane potential dependent probe, 3,3′-dipropylthiadicarbocyanine iodide [DiSC_3_(5)]. Upon permeabilization and disruption of the membrane, the membrane potential is dissipated, and DiSC_3_(5) is released into the medium causing an increase in fluorescence. PAAG treatment depolarized the membrane of *P. aeruginosa* persister cells in a dose dependent manner (**Figure 6**). All concentrations of PAAG tested showed significantly greater ability to depolarize the cytoplasmic membrane compared to colistin (*P* < 0.0001), suggestive of PAAG’s mechanism by which it completely eradicated the CCCP-induced persister cells (**Figure 4**).

Patients with relapsing chronic infections including those with CF, chronic obstructive pulmonary disease (COPD), bronchiectasis and immune compromise are often plagued by infections from both Gram-negative and Gram-positive pathogens. The variety of infecting organisms and antibiotic resistance status have resulted in a wide range of different therapeutic needs (Ventola, 2015). Currently, treatment of infections caused by persisters requires prolonged and repeated exposure to strain-specific antibiotics, with elevated risk of generating genetic antibiotic resistance and relapse (Zindani et al., 2006). Tobramycin and aztreonam, drugs that are currently being used to treat recalcitrant CF infections, must be given only periodically to minimize doses that can ultimately cause harmful side effects (Mearns, 1972; Zindani et al., 2006). A recent 10-year safety study on clarithromycin and azithromycin link their use to increased deaths in immune compromised individuals with heart conditions (Spoering and Lewis, 2001; Krahn et al., 2012). Colistin, a last resort antibiotic to treat chronic infcetions, has been prescribed with increasing frequency, out of necessity, and is often associated with side-effects of nephrotoxicity and neurotoxicity (Falagas et al., 2005; Honoré et al., 2014; Miano et al., 2018). A recent study published sheds further evidence into the cytotoxicity of polymyxins as they were found to induce apoptosis in human lung epithelial cells in a concentration and time dependent manner (Ahmed et al., 2017). To assess if PAAG also displays cytotoxicity the LDH assay was used. LDH release, from the lung epithelial cell line A549, was assayed using a range of concentrations of PAAG (65–1,000 μg/mL). Limited release of LDH was observed at 1,000 μg/mL indicative of minimal cytotoxic effects (**Figure 7**). Although this result is limited to one cell line the proposed method of drug delivery, inhalation, made the choice of A549 cells particularly relevant. However, studies are still ongoing, currently no significant decrease in activity has been observed against clinical isolates of *Pseudomonas* in artificial sputum media (data not shown).

PAAG has shown activity against both Gram-positive and Gram-negative pathogens (Narayanaswamy et al., 2017; Wood, 2017) despite their structural differences. Though this preliminary study is limited to persisters formed in a single bacterial species, *P. aeruginosa*, this opportunistic pathogen has served as the main model for persister studies as it produces high levels of drug-tolerant persister cells. PAAG as a membrane-acting glycopolymer eradicates *P. aeruginosa* persister cells *in vitro* at doses that show minimal cytotoxicity to human epithelial lung cells even at the highest dose tested (1 mg/mL; **Figure 7**). Studies are ongoing to better understand the broad spectrum of PAAG’s activity against other important pathogens in chronic lung infections *in vitro* and how PAAG’s activity translates to an *in vivo* setting. The findings of this study show strong potential for this new class of glycopolymer drugs in the fight against chronic lung pathogens and antibiotic resistance.

## Author Contributions

VN study design, formal analysis, validation, visualization, writing – original draft, writing – revisions, review, and editing. VN, LK, and KD investigation and methodology. ST formal analysis, initial study design, and writing – first draft. WW, SB, and ST conceptualization, funding acquisition, resources, writing – review and editing. AL study design, writing – final revisions, editing, and review.

## Conflict of Interest Statement

VN, LK, KD, ST, AL, SB, and WW are paid employees of Synedgen. ST, SB, and WW have ownership and patents affiliated with Synedgen, and SB and WW are board members. The glycopolymer used in this study is protected by US Patent number 8,119,780 B2 and others pending in development of drug products to treat lung infections and related indications. The potential conflicts noted have not impacted or influenced the findings of this manuscript.
